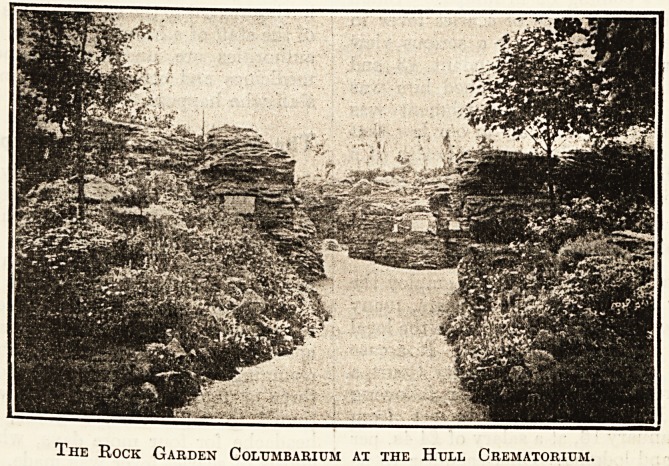# Cremation: Its Cost and How It Is Carried out

**Published:** 1912-04-06

**Authors:** 


### CREMATION.

### Its Cost and How it is Carried Out.

The method of disposal of the dead by burning has of late years received an increasing amount of attention, and the movement initiated by Sir Henry Thompson in 1874 has made considerable strides since then, though the progress cannot be said to have been very rapid. In this article we shall not dwell upon the advantages of cremation, but we shall endeavour to give concisely practical information concerning the steps which must be taken by the friends of the dead person and the legal and other formalities which have to be gone through, as well as to give some idea of the cost of cremation.

As this question of cost is one which comes to the fore at an early stage in the proceedings, it will be perhaps well to consider this point first. We may safely surmise that a much larger number of cremations would take place yearly were it not for the prevalent notion that this process is an expensive one, and one involving an excessive and annoying series of formalities. The assiduous attentions of interested undertakers at such times may also be supposed to influence the relatives, and tend to persuade them to take the path of least resistance, ending in earth burial, even when there exists no prejudice against cremation.

The cost of cremation, even at the present time, is less than that of a burial of the same class, and when cremations become more frequent, as they undoubtedly will, the working expenses at the crematoria will be proportionately less and the cost of each cremation will be very materially reduced. Under present conditions the furnaces have to be reheated practically for every single cremation.

The fee for the actual cremation, including the use of chapel and attendance, may be put at from four to five guineas. In several provincial crematoria under the control of the municipal authorities the fees are greatly reduced for local residents or in the case of poverty, and in many places only two guineas is charged. In Hull the fee is one guinea for residents in the city.

Owing to these variations in the charges it is impossible to give exact estimates, for naturally such additional charges as those for the chaplain's services, for the urn for the ashes, or for decoration of the chapel, will also vary, or may even be included in the cremation fee, as is the case in certain provincial centres.

The expense of the funeral itself—that is to say, the costs incurred from the time of death till the undertakers have transported the body to the cemetery or the crematorium—will be nearly the same in both cases. For if there be some extra expense in connection with the certificates required, there is, on the other hand, no occasion to spend any considerable sum on the coffin, which must, indeed, conform with certain regulations and be made of light wood in the simplest style, with easily removable brass fittings, if any.

The Crematorium at Leeds.

It is, therefore, quite fair to compare the expenses which will be probably incurred after the body has been placed on the catafalque at the crematorium in the one case, with the costs payable at an ordinary cemetery in the other case of earth burial. Taking an average of the charges made at six London cemeteries for burial in ordinary ground, we find that the cost of a private earth grave is about £5 5s., and that the interment fee averages about £2 10s. In addition, the cost of a headstone or kerbs must be allowed for; in fact, this is compulsory at many cemeteries. For this twelve guineas is an exceedingly moderate estimate. Thus we arrive at a total expense of £20 7s. At a crematorium the fee for the incineration will not be more than five guineas, to which must be added half a guinea for the urn. If we include the optional, but commonly incurred, expense of purchasing a niche in the columbarium for the reception of the urn, a further charge of, say, four guineas must be allowed for. With an additional half-guinea for the services of a chaplain (also optional), the cost of a cremation comes, therefore, to about £10 10s. Expenses in both forms of disposal of the dead will naturally vary enormously in individual cases, but when cases of the same class

Plan of the Leeds Crematorium.

are compared, the advantage in the matter of cost will be found to lie with cremation. Several firms advertise to carry out a cremation completely at inclusive charges ranging from eight to thirteen guineas and upward—that is to say, including the funeral expenses from the house to the crematorium, as well as all costs incurred until the ashes are placed in an urn.

Regulations and instructions are issued by the authorities controlling crematoria, and as these are to a large extent founded on the legal requirements, we may now conveniently give an epitome of the law on this subject. First, it is interesting to note that the Common Law does not recognise any property in the dead body of a human being, so that a person cannot dispose of his body by will, except in the one case specially provided by statute—namely, for anatomical research. Likewise any direction in a will as to how or where the testator's body is to be disposed of has no binding force in law, and may be disregarded with impunity; indeed, more often than not the will is not read until after the funeral. It is open to the testator to make a gift to an executor depend on the way in which his body is disposed of, and in such a case he should acquaint his executor with what he has done. It must be remembered that it is provided by a rule in the statutory regulations made by the Home Secretary that the Medical Referee shall not allow cremation to take place if it appears that the deceased left a written direction to the contrary. Except in this case, cremation is as lawful a manner of disposing of a dead body as burial in the earth; moreover, the word "bury" is not to be taken as confining the executor to earth burial.

The law on the subject of cremation is embodied in the Cremation Act of 1902 and in Statutory Rules and Orders made by the Home Secretary under Section 7 of the Act. The clauses of the Act relate to the powers of burial authorities and the construction of crematoria, and include a description of certain offences in connection with the declarations and certificates. The incumbent of a parish is absolved from any obligation to perform a funeral service at a cremation of a parishioner, and the scale of fees is to be approved by the Local Government Board, which charges or fees shall be deemed to be part of the funeral expenses of the deceased.

It will be seen from the rules that when a cremation is to be performed there is a far more satisfactory investigation into the cause of death than in the ordinary course of death certification, the imperfections of which are well known. Indeed, we believe that something like eight thousand bodies are buried yearly in England and Wales without any medical certificate whatever. Cremation does, as its advocates insist, provide a much more effective safeguard against foul play than in the case of ordinary earth burial, and is in this way a real protection of society against crime.

The certificates required under the statutory regulations are readily procurable from the cremation authorities—in London, from the Company's offices at 324 Regent Street, W.—and with them is also issued a detailed list of instructions which will show clearly the steps required to be taken. The form of application for cremation has to be first filled up, and it must be remembered that this requires to be supported by a statutory declaration, made on the same form, that all the particulars given are true. This may be made before a Commissioner of Oaths or a Justice of the Peace, who need not be a Justice for the county or place where the declaration is made. The Commissioner's fee is 1s. 6d., or, if he has to attend the house of the declarant, he is entitled to an additional 6s. 8d.

Another point to be noted is that the certificate of registry of death must be produced and that neither a certificate of notification of death nor a certified copy of an entry in the register of deaths is sufficient. The medical certificates are too long to be reproduced here, but they require full details of all the circumstances of the illness or accident causing the death, and end with a declaration that there is no circumstance known to the subscriber "which can give rise to any suspicion that the death was due wholly or in part to any other cause than disease (or accident), or which makes it desirable that the body should not be cremated." The confirmatory medical certificate will usually not cost more than one guinea.

Immediately after the death an undertaker should be instructed that cremation is intended and a suitable coffin ordered. The certificates necessary are very often obtainable from the undertaker himself. Then the declaration on Form A must be filled up by an executor or the nearest surviving relative, or, in accordance with rule No. 7, satisfactory reasons given if the application is made by someone else. This must then be forwarded with the registrar's certificate for burial to the Medical Referee of the cremation authority or company. The certificate on Form B, signed by the deceased's medical attendant, is to be sent to the medical practitioner who is to give the confirmatory certificate on Form C, and who has to be of a certain standing in the profession.

If an inquest has been held or a post-mortem examination made, different certificates (E or D) will be used instead of Form C. The authority to cremate will be given by the Medical Referee on the receipt of the above certificate.

As regards the usual funeral arrangements these are, of course, a matter for individual arrangement and taste, and need differ in no respect from those of an earth burial. But if any elaborate outer coffin and furniture be ordered, arrangements must be made with the undertakers for removing these forthwith from the crematorium premises. A coffin made of cotton wood, thin pine, three-ply wood, elm, or some readily combustible material is far preferable, for authorities usually deprecate strongly the use of an outer coffin, which would have to be removed before incineration. The only metal case allowable is a zinc one, as this metal is readily consumed. Certain other points, of interest only to the undertakers, need not here be mentioned.

Upon arrival of the body at the chapel, if there is a funeral service, this is at once proceeded with, the coffin resting the while upon the catafalque which is generally supported on a marble daïs. At the proper moment the coffin is made to glide noiselessly by mechanical means through the small doors into the cremating chamber beyond.

The shell or coffin actually containing the body is under no circumstances opened after it has passed into the charge of the authority; friends may follow the body into the incinerating chamber, but they are not allowed to inspect the actual process of cremation. The incineration generally occupies about an hour and a half and the ashes are then gathered together and handed to the relatives. Care is taken to maintain them intact and pure for this purpose.

Caskets and urns of great variety are now easily obtained for the reception of the ashes; these may be, at will, either removed by the friends or placed in niches in the columbarium, which may be rented in perpetuity. If desired, private tombs or mausoleums may be erected, or, on the other hand, the ashes may be left to be decently buried by the authorities. It is obvious that in cases where death has occurred at a distance from the family grave or vault, cremation offers a convenient means of overcoming the difficulties and great expense of transporting a dead body.

There are now in working order thirteen crematoria in various parts of the country, and last year eight hundred and forty cremations took place. In the order of the duration of their existence the following is a list of their locations: Woking, Manchester, Glasgow, Liverpool, Hull, Darlington, Golder's Green, Leicester, Birmingham, Leeds, Ilford, Bradford, and Sheffield. There is, we understand, a good prospect of the establishment of a crematorium at Bristol in the near future.

In spite of these facilities cremation is but slowly progressing, especially among the poorer classes of the community. To these the idea still arouses a considerable amount of prejudice, which has resisted all the inducements offered in the shape of greatly reduced cost, as at Ilford, for among the poor economy in funeral expenses is never held to be good form. But the number of persons of scientific, literary, or social distinction cremated in Great Britain is out of all proportion to what would be a normal average at the present stage, and it is interesting to note that within the last year Westminster Abbey has received for the first time the ashes instead of the body of the distinguished dead. This precedent will go far in solving the difficult problem of providing more space for interment in that honoured resting-place.

The Rock Garden Columbarium at the Hull Crematorium.

It may be of interest to some to know that there is on view at the Museum of the Royal Sanitary Institute in Buckingham Palace Road a large model of one of the cremation furnaces in use at Golder's Green.

Anyone who desires to insure cremation at death can do so conveniently by becoming a member of the Cremation Society of England, for the payment of six annual subscriptions of one guinea (or five guineas down) carries the privilege of being cremated at any British crematorium *without fee*, and a certificate is given to that effect.

## Figures and Tables

**Figure f1:**
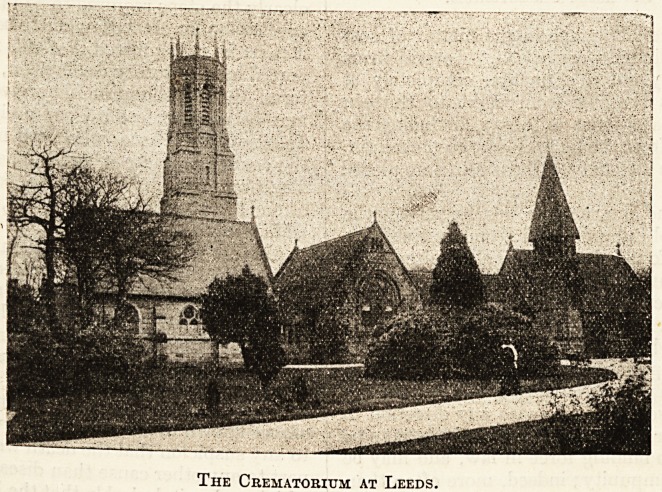


**Figure f2:**
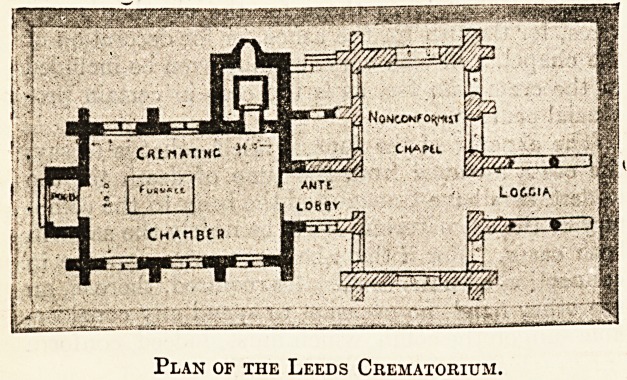


**Figure f3:**